# Digital Transformation in Healthcare and Nephrology

**DOI:** 10.7759/cureus.102297

**Published:** 2026-01-26

**Authors:** Diogo A Domingos, Ana C Martins, Patrícia Matias, Célia Gil

**Affiliations:** 1 Nephrology Department, Hospital de Santa Cruz, Lisbon, PRT

**Keywords:** artificial intelligence, clinical decision support systems, digital transformation, electronic health records, interoperability, mhealth, telemedicine

## Abstract

The recent rise in the use of artificial intelligence (AI) in medicine has generated considerable enthusiasm. However, the emphasis on advanced tools, such as large language models, obscures the challenge of incomplete digital transformation in everyday clinical practice. In nephrology, as in other specialties, workflows remain heavily reliant on manual tasks and are often fragmented and non-interoperable. This situation not only adds a significant bureaucratic burden to clinicians but also hampers the development of high-quality and structured data, necessary to power AI models. In this review, we argue that a human-centered approach to digitalization is essential for unlocking AI’s full potential in healthcare. By applying service design principles and prioritizing the needs and workflows of end-users, it becomes more probable that valuable digital health tools will be developed. This is best achieved through the active co-creation of these platforms with both healthcare professionals and patients, establishing true interoperability via common data standards, and designing systems that enable the capture of structured information for primary and secondary purposes, such as clinical research and quality improvement.

We view nephrology as uniquely positioned to serve as a "living lab" for this digital transformation. The specialty manages a diverse patient population, including those with chronic kidney disease, transplant recipients, and individuals with rare diseases, all requiring complex and long-term care. By initially establishing a robust digital infrastructure to address urgent clinical challenges, we can lay the groundwork for AI to be deployed safely and effectively, thereby enhancing patient care.

## Introduction and background

Since the public release of large language models (LLMs), like ChatGPT, in late 2022, the number of both scientific and media publications on the applications of artificial intelligence (AI) in medicine has increased significantly [1]. Nephrology was no exception to this trend, with growing interest in how these technologies could be applied to the specialty.

Although AI in the form of LLMs has received most of the media attention, other tools, such as computer vision and machine learning, have been in use for much longer. For years, machine learning models have been designed to detect and predict the progression of chronic kidney disease (CKD) and the onset of acute kidney injury [2]. Computer vision has also shown promise in analyzing renal pathology images [3]. Of course, LLMs keep their own distinct role in the medical field, from enhancing communication with patients by simplifying complex topics to improving access to medical education for trainees [4]. While many papers have reviewed the importance of updating medical practice to incorporate these AI tools, others have questioned the actual benefits they can offer and the potential threats they pose to medical professionals [5].

The debate about the future of AI in nephrology is unavoidable, but it must not overshadow a more immediate challenge: the incomplete digital transformation of routine clinical practice. Workflows continue to depend heavily on manual processes, creating a significant bureaucratic burden for healthcare professionals and diverting their attention from patient care. This issue directly limits the potential of AI: powerful machine learning models rely on large amounts of high-quality, structured, and accessible digital data, which our current analog or fragmented systems fail to provide. Therefore, while we can and should promote AI and digitalization together, we must recognize that without a solid digital infrastructure, AI’s true potential will remain limited to research environments rather than serving as a practical tool in the daily management of patients.

In this narrative review, we will explore the elements of digital transformation and present a practical framework for its realization in nephrology. We will begin by defining what digital transformation in healthcare entails and how it can help alleviate the bureaucratic burden faced by clinicians. Next, we will emphasize the importance of service design and co-creation, highlighting the need to involve healthcare professionals in the development of digital tools. We will then evaluate the technical framework required for success, with a focus on interoperability and the primary and secondary uses of health data.

## Review

Digital transformation in healthcare

Digital transformation can be defined as a process that strategically combines information, computing, and connectivity technologies to drive significant improvements within an organization or entity [6]. When applied to the healthcare setting, it refers explicitly to the integration of digital technologies, such as AI, electronic health records (EHRs), telemedicine [7], mobile health (mHealth) [8], and big data [6] into healthcare practices to improve both workflow efficiency and patient care.

The slow pace of digital transformation in healthcare stems from resistance across multiple fronts. First, healthcare professionals often oppose the adoption of information technology, viewing it as an administrative burden and a threat to their professional autonomy, and sometimes lack the skills and competencies to utilize digital tools [9]. At the organizational level, providers may have few incentives to implement digital systems or share data due to concerns about competitiveness. Significant barriers also include high initial and ongoing costs, productivity losses during the transition, and concerns about future technological obsolescence [10]. Patients are also a key source of resistance, often reluctant to disclose highly personal health information, especially when they perceive it as sensitive or distrust the organizations that collect it.

Lastly, developing and deploying health information systems is often problematic. Many products are not user-friendly, requiring lengthy data entry that disrupts clinical practice rather than supporting it. The lack of user-centric design is especially evident in the structure of many modern electronic systems, including EHRs. Often, they are not truly innovative tools, but rather digitized versions of the paper-based systems clinicians have used for decades. While these digital records offer the advantage of being widely and instantly accessible across a health system network, they fall far short of their potential. Many systems lack robust and intelligent features such as automated alerts for critical laboratory or microbiology results, the ability for automatic data transcription to reduce manual entry, or genuine interoperability that allows for seamless transfer of patient data between different electronic systems and providers. This forces clinicians to act as manual integrators of fragmented information, and the inefficiency of these systems consumes a significant portion of healthcare professionals’ working hours, sometimes with more time spent on data entry in EHRs than on any other work-related activity [11]. Consequently, studies indicate that healthcare professionals report an increased workload with some digital tools, partly caused by the failure of EHRs to accommodate the different approaches to tasks that physicians adopt in their practice [12].

The daily routine of healthcare professionals is filled with administrative duties such as transcribing medication lists, manually calculating clinical scores, or filling out standardized forms. It is precisely these series of repetitive tasks that would benefit most from meaningful digital transformation. By redesigning digital workflows to manage these routine processes, the bureaucratic burden on clinicians can be significantly reduced. This not only alleviates burnout and decreases the risk of human error but also frees up valuable time and cognitive resources, enabling professionals to concentrate on clinical reasoning, decision-making, and direct patient interaction.

Digital health tools design: co-creation and interoperability

To address the issues of poor usability and low adoption, the development of digital health tools must transition from a technology-first approach to a human-first one. This is the core principle of Service Design, a methodology for platform creation that emphasizes delivering optimal user experiences. In healthcare service design, professionals act as both providers and primary users in the environments where care is delivered.

Co-creation

When developing digital health tools, a user-centered approach, which states that the value of any digital tool is determined by its ability to meet the real-world needs of its users, has a higher probability of success. This user-centered philosophy is best realized through co-creation, an active and ongoing partnership that involves a multidisciplinary team of healthcare professionals (such as nephrologists, nurses, and pharmacists), technical experts, and patients in the development process [13]. Clinicians are experts on clinical workflow, safety, and efficiency needs. At the same time, patients bring their lived experience of disease and are best suited to guide the design of practical and engaging tools for home use [14]. By involving both groups (including patient advocates and associations) from the initial brainstorming to the final implementation, co-creation ensures the product is technologically sound, clinically relevant, and practically usable, thereby increasing the likelihood of successful adoption.

Behavioral Interventions in Digital Health

Behavior science employs findings from psychology and other social sciences to understand and influence individual behavior. Applying behavioral theories in the development of digital tools can be valuable not only for improving user engagement with this technology but also for actively guiding both clinicians and patients towards actions that lead to better health outcomes [15]. Models such as the Capability, Opportunity, Motivation and Behavior (COM-B) [16] model and the Behavior Change Wheel [17] are structured guidelines for designing these interventions, such as "nudges." An example of this kind of intervention is the use of digital platforms in supporting self-management routines for patients with chronic conditions, such as CKD [18] or heart failure [19].

Health Data and Interoperability

During daily clinical practice, healthcare professionals frequently record data on digital platforms. Often, this data is documented in plain text. This narrative format effectively serves the primary use of data, direct patient care, by allowing nuance and detailed explanations of clinical plans, which ensures a clear understanding for other healthcare professionals who may later assume care of the patient. However, much of the potential value of health information is lost because this unstructured data is complex for computer systems to analyze at scale. This poses a challenge to using health data beyond direct clinical care (secondary use), for purposes like clinical research, public health monitoring, quality improvement initiatives, and, importantly, for training the machine learning models that power advanced AI tools [20].

To unlock the potential of the vast amounts of data generated in healthcare settings, clinical information systems must facilitate the capture of structured data. This involves recording key clinical concepts using standardized terminologies and codification - e.g., Systematized Nomenclature of Medicine Clinical Terms (SNOMED-CT) for diagnoses, and Logical Observation Identifiers Names and Codes (LOINC) for lab tests [21] - in designated fields. The ideal EHR would not force a choice between these two formats but would allow clinicians to write their narrative notes while capturing the essential information as structured data in the background, an approach that would ensure that data remains understandable for human communication while also being clean, organized, and immediately ready for analysis by machine-learning tools.

Another important aspect is interoperability, which is the ability of different digital systems to communicate and exchange data seamlessly between them. According to the report State of Digital Health 2024, only 15% of European Union countries have digital healthcare architectures that enable this cross-sharing of information [22]. Interoperability has benefits by allowing safer patient care, improved efficiency, and cost savings by reducing the need for repeated medical exams. Finally, it provides better data for research and public health, making it easier for scientists to combine large sets of information to advance medical knowledge and monitor population health [23].

Nephrology as a living lab for digital transformation

In the context of digital transformation, a living lab is a real-world clinical environment that acts as a collaborative platform for innovation, where the methodologies previously discussed are implemented: new digital tools are developed using a user-centric design approach, refined through active co-creation between developers, clinicians, and patients, and enhanced with features based on behavioral insights. This enables teams to evaluate, within routine clinical care, whether a new tool is not only functional but also practical, usable, and capable of positively changing workflows and health outcomes. Success can be measured through a combination of process metrics (such as adoption rates or time saved on documentation), clinical indicators, and patient-reported experience measures and outcomes [24].

A nephrology department is well-positioned to serve as a living lab for digital transformation because it manages a broad spectrum of conditions, ranging from common and chronic to complex and rare diseases. For patients with CKD, the patient journey is lengthy and involves critical, life-altering decisions that require clear communication and shared decision-making among patients, their families, and clinicians. Furthermore, managing end-stage renal disease heavily depends on behavioral change, with dietary and lifestyle measures being essential for reducing complications. The growing importance of home therapies highlights the need for digital tools that facilitate remote monitoring and patient engagement.

Nephrology is at the forefront of managing high-need populations, such as kidney transplant (KT) recipients and patients with rare autoimmune or genetic diseases, which present distinct but related challenges well-suited for digital intervention. For transplant recipients, lifelong immunosuppressive therapy is required, making strict medication adherence vital to prevent graft rejection. For rare diseases, gathering high-quality, longitudinal data for clinical investigation is difficult, but crucial. Digital platforms offer a powerful and unified solution to these challenges: they can be co-created with patients to support complex behavioral requirements like adherence, while simultaneously creating the infrastructure to collect standardized, longitudinal data. This data can then be used to track trends, alert clinical teams to potential issues, and populate multi-center registries to accelerate clinical research. This makes the nephrology department an ideal environment to explore how a comprehensive digital strategy can transform care, from improving daily clinical practice through enhanced medical communication and remote management with telemedicine and mHealth, to supporting patient self-care using nudges and behavioral change methodologies.

Telemedicine and mHealth

Over recent years, there has been a push for the adoption of home dialysis, including both peritoneal dialysis (PD) and home hemodialysis, due to its lifestyle benefits and lower costs [25]. A nephrology unit with an extensive home dialysis program offers an ideal setting to innovate in telemedicine. Patients on home therapies are already engaged in self-care, making them excellent partners for testing new digital tools. Remote monitoring tools, such as wearable devices and smartphone applications, enable early detection of complications and help reduce hospitalizations and costs [26,27].

mHealth platforms facilitate continuous data collection, which, when reviewed by healthcare professionals, can reduce the need for frequent in-person consultations. In one study involving patients in PD, a mobile app was implemented to record blood pressure, weight, diet, PD prescription, medications, sleep status, and PD catheter exit-site condition [28]. This data was then analyzed in real-time by an AI agent that alerted the healthcare team upon detecting any abnormalities. The intervention had a positive impact on patient-reported quality of life and treatment adherence, resulting in improved clinical outcomes, including better management of hypertension, anemia, and mineral bone disease [29].

Big Data and Clinical Investigation

Each hemodialysis session generates hundreds of data points, and patients with CKD and KT recipients have frequent lab tests and clinical encounters over many years. The data collected is being used to develop machine learning algorithms capable of predicting hemodialysis complications [30], improving the early detection of CKD [31], and assessing the risk of progression for kidney failure in this population [32].

Big data can also facilitate advances in personalized care, such as its potential role in adjusting immunosuppressant doses for KT recipients. Predictive algorithms that integrate demographic, concurrent medication, and past drug level monitoring can assist in personalized dosing recommendations, helping clinicians achieve stable and adequate immunosuppressive drug levels more quickly and accurately than traditional protocols, thereby minimizing the risks of rejection or drug toxicity [33].

Customized EHRs for Rare Diseases

As a specialty that manages rare autoimmune and genetic diseases, nephrology can lead the development of digital infrastructure that improves patient care and clinical investigation in this field. Instead of relying on a generic EHR, a department can pilot customized modules that record data points specific to a rare disease (e.g., genetic or autoimmune activity markers, symptom scores, or other disease activity measures). This approach guarantees that high-quality, research-grade, structured data is gathered as part of routine clinical care. These customized EHR modules could then be designed to automatically feed into a secure national or international registry dedicated to a particular rare disease, aiding clinical research studies and trial recruitment, which is often a significant challenge for rare conditions. The need for such platforms is already being discussed in other specialties that deal with rare diseases, such as rheumatology [34] and oncology [35]; it is now essential to bring this conversation to the forefront within nephrology.

Clinical Decision Support Systems (CDSS)

CDSS are digital tools that provide clinicians with timely and patient-specific recommendations at the point of care, enhancing clinical decision-making. A CDSS can utilize data from the EHR, such as laboratory results, to provide the clinician with advice on managing patients. Although nephrologists believe that using CDSS would be beneficial in managing CKD, the adoption of these tools remains low, primarily due to a lack of awareness about their existence [36]. In primary care settings, a CDSS can be used to identify at-risk patients, allowing for early initiation of prognosis-modifying therapies, prophylactic medication, or referral to a specialized nephrology center [37].

Medical Communication and Behavioral Change

Nephrology practice is filled with difficult conversations about life-altering decisions, such as starting dialysis, choosing a modality, or transitioning to palliative care. Digital tools can be developed to enhance this shared decision-making process by allowing continuous access to complementary and simplified information about procedures and complex diagnoses, an element that is often lacking [38].

Digital tools also play a role in personalized, behavior-change-directed interventions. For patients on dialysis who require significant lifestyle, dietary, and activity changes, "nudges" in the form of customized reminders can encourage positive behaviors that reduce complications and improve quality of life. Digital platforms are effective in implementing these strategies and have been shown to improve physical activity and nutrition in maintenance hemodialysis [39]. However, it is essential to continuously evaluate the efficacy of these nudges, allowing for the integration of impactful interventions into clinical practice while discarding those that are ineffective and may contribute to alert fatigue.

Clinical Audits

Clinical audits are an essential part of quality review and continuous improvement in healthcare systems; however, the significant time, resources, and effort required often hamper their implementation [40]. Digital tools can assist by automating the audit process, increasing the coverage of audited cases from a small sample to nearly 100% of all events, and enabling teams to identify and address deficits in training or knowledge promptly. In nephrology, a similar approach can be employed to develop a live dashboard of key indicators, such as infection and hospitalization rates, thereby facilitating early detection of problems and interventions.

Sustainability

Nephrology imposes a significant environmental burden, especially from resource-intensive therapies like hemodialysis, which consumes large quantities of water and energy while generating considerable plastic waste [41]. Digital health technologies offer a promising pathway towards "green nephrology." For instance, employing smart sensors in dialysis units allows real-time monitoring of resource consumption. Such data can be used to test and evaluate interventions, such as optimizing water purification cycles, implementing energy-saving protocols for equipment, or improving waste segregation, to reduce the environmental footprint.

## Conclusions

Before the full potential of complex algorithms of AI can be realized in medicine, we must first address the incomplete digital transformation of our daily clinical practice. The solution lies not solely in technology, but in a human-centered approach built on service design, co-creation with clinicians and patients, and the development of interoperable and structured data ecosystems.

In this review, we have demonstrated that nephrology, with its unique mix of managing chronic diseases, complex home therapies, transplantation, and rare diseases, could function as an ideal living lab to lead these changes. By focusing on developing tools that address immediate issues such as bureaucratic burdens and fragmented data, we not only enhance patient care and clinician workflows but also lay a solid digital foundation for the AI revolution in healthcare to be established safely and effectively.
